# Supplementary material for: Modeling glioblastoma heterogeneity as a dynamic network of cell states
Source: Mol Syst Biol. 2021 Sep 16;17(9):e10105. doi: 10.15252/msb.202010105 (PMC8444284; doi:10.15252/msb.202010105)
Supplement: Supplementary file 6 — Source Data for Figure 5 [file MSB-17-e10105-s004.zip › Figure5A_sourcedata/GSEA_3071/nosigdb_stateII.GseaPreranked.1622795151171/RICHARDS_NEFTEL_CELL_2019_NPC2.html]

Details for gene set RICHARDS\_NEFTEL\_CELL\_2019\_NPC2[GSEA]

|  || Dataset | state23071 |
| Phenotype | NoPhenotypeAvailable |
| Upregulated in class | na\_neg |
| GeneSet | RICHARDS\_NEFTEL\_CELL\_2019\_NPC2 |
| Enrichment Score (ES) | -0.71656585 |
| Normalized Enrichment Score (NES) | -2.8947415 |
| Nominal p-value | 0.0 |
| FDR q-value | 0.0 |
| FWER p-Value | 0.0 |
Table: GSEA Results Summary

  

Fig 1: Enrichment plot: RICHARDS\_NEFTEL\_CELL\_2019\_NPC2      
 Profile of the Running ES Score & Positions of GeneSet Members on the Rank Ordered List

  

| PROBE | GENE SYMBOL | GENE\_TITLE | RANK IN GENE LIST | RANK METRIC SCORE | RUNNING ES | CORE ENRICHMENT || 1 | LBH |  |  | 182 | 0.898 | -0.0086 | No |
| 2 | RND3 |  |  | 860 | 0.482 | -0.1556 | No |
| 3 | STMN1 |  |  | 1249 | 0.387 | -0.2353 | No |
| 4 | ATP1B1 |  |  | 2433 | -0.275 | -0.5149 | No |
| 5 | CEP170 |  |  | 2737 | -0.324 | -0.5763 | No |
| 6 | MLLT11 |  |  | 2760 | -0.328 | -0.5686 | No |
| 7 | PAK3 |  |  | 2839 | -0.343 | -0.5740 | No |
| 8 | MIAT |  |  | 2996 | -0.371 | -0.5974 | No |
| 9 | DYNC1I1 |  |  | 3221 | -0.433 | -0.6350 | No |
| 10 | TUBB2A |  |  | 3554 | -0.572 | -0.6936 | Yes |
| 11 | FNBP1L |  |  | 3615 | -0.614 | -0.6837 | Yes |
| 12 | CD24 |  |  | 3624 | -0.620 | -0.6608 | Yes |
| 13 | KIF5A |  |  | 3631 | -0.627 | -0.6370 | Yes |
| 14 | DPYSL5 |  |  | 3668 | -0.659 | -0.6194 | Yes |
| 15 | TAGLN3 |  |  | 3751 | -0.729 | -0.6103 | Yes |
| 16 | SOX4 |  |  | 3797 | -0.786 | -0.5897 | Yes |
| 17 | NREP |  |  | 3805 | -0.794 | -0.5596 | Yes |
| 18 | KIF5C |  |  | 3813 | -0.800 | -0.5292 | Yes |
| 19 | DDAH2 |  |  | 3895 | -0.936 | -0.5115 | Yes |
| 20 | PKIA |  |  | 3950 | -1.056 | -0.4823 | Yes |
| 21 | SOX11 |  |  | 3977 | -1.147 | -0.4426 | Yes |
| 22 | TCF4 |  |  | 3984 | -1.173 | -0.3970 | Yes |
| 23 | NFIX |  |  | 4004 | -1.262 | -0.3510 | Yes |
| 24 | NFIB |  |  | 4007 | -1.310 | -0.2989 | Yes |
| 25 | TUBB3 |  |  | 4009 | -1.334 | -0.2455 | Yes |
| 26 | DPYSL3 |  |  | 4012 | -1.349 | -0.1918 | Yes |
| 27 | DLX5 |  |  | 4090 | -2.619 | -0.1056 | Yes |
| 28 | DCX |  |  | 4091 | -2.671 | 0.0017 | Yes |
Table: GSEA details [plain text format]

  

Fig 2: RICHARDS\_NEFTEL\_CELL\_2019\_NPC2: Random ES distribution      
 Gene set null distribution of ES for **RICHARDS\_NEFTEL\_CELL\_2019\_NPC2**
